# Challenges in performing clinical research on herbal products – experiences from Switzerland and the UK

**DOI:** 10.3389/fphar.2026.1766381

**Published:** 2026-01-26

**Authors:** Angélique Bourqui, Chantal Csajka, Michael Heinrich, Clara Podmore, Catherine Simpson, Jeanne Trill, Merlin Willcox, Pierre-Yves Rodondi

**Affiliations:** 1 Faculty of Science and Medicine, Institute of Family Medicine, University of Fribourg, Fribourg, Switzerland; 2 Center for Research and Innovation in Clinical Pharmaceutical Sciences, University Hospital and University of Lausanne, Lausanne, Switzerland; 3 School of Pharmaceutical Sciences, University of Geneva, CMU, Geneva, Switzerland; 4 Institute of Pharmaceutical Sciences of Western Switzerland, University of Geneva, University of Lausanne, Lausanne, Switzerland; 5 Pharmacognosy and Phytotherapy, UCL School of Pharmacy, University London, London, United Kingdom; 6 Department of Pharmaceutical Sciences and Chinese Medicine Resources, Chinese Medicine Research Center, College of Chinese Medicine, China Medical University, Taichung, Taiwan; 7 Southampton Clinical Trials Unit, University of Southampton Clinical Trials Unit MP131, Southampton General Hospital, Southampton, United Kingdom; 8 Primary Care and Population Science, University of Southampton Faculty of Medicine, Aldermoor Health Centre, Southampton, United Kingdom

**Keywords:** clinical trials, GMP, herbal products, quality, regulation

## 1 Introduction

Use of herbal food supplements (HFS) is popular in Europe (Serafini et al., 2012); with an annual market value of $7.5 billion (Virtue Market Research, 2025). Use in Switzerland is much more widespread than in the UK (Heinrich et al., 2023). HFS are used for a wide range of indications, from general health promotion to the management of acute or chronic disease (Ng et al., 2022). Despite their widespread use, and calls for high-quality evidence assessing their efficacy and safety, clinical trials remain limited (Jalil et al., 2025) – and not always for the reasons one may suspect.

Clinical trials intended to assess herbal products for medical indications are subject to the same regulatory requirements as those applied to pharmaceutical drugs (European Medicines Agency, 2022). In addition to ethical approval, the study must receive authorization from the relevant drug agency, which evaluates the quality and safety of the investigational medicinal product (IMP), following the same strict standards used for pharmaceutical drugs.

Meeting these requirements for herbal products can be challenging, depending on whether they are categorised as herbal medicinal products (HMPs) or herbal food supplements (HFSs). HMPs are regulated by national drug agencies such as Swissmedic (in Switzerland) or the Medicines and Healthcare products Regulatory Agency (MHRA, in the UK), which assess their safety, quality, and efficacy prior to marketing (European Parliament and the Council of the European Union, 2004). HMPs are thus more likely to meet the requirements for use in clinical trials (Heinrich et al., 2024). In contrast, HFSs fall under food regulations and are not subjected to any quality or safety assessment prior to commercialization (Office fédéral de la sécurité alimentaire et des affaires vétérinaires, 2024; Rocha et al., 2016; Saper et al., 2004). The uncertain quality of HFSs makes conducting clinical trials more challenging. Grounded in practical experience, this article aims to outline the specific challenges investigators currently face in meeting regulatory requirements for clinical trials involving HFSs as the IMP.

## 2 A brief overview of general requirements from European drug agencies

The European directive 2001/83/EC describes safety, efficacy, and quality requirements for IMPs (European Medicines Agency, 2022). The pharmaceutical quality dossier required by drug agencies must include a detailed description of the manufacturing processes for both the active substance (in this case, generally a mixture of substances intended to exert a pharmacological, immunological or metabolic action) and the finished product. It must also outline the procedures used to guarantee product quality, purity, and stability. Manufacturers are required to produce medicinal products in accordance with Good Manufacturing Practice (GMP) standards and to be GMP-certified. GMP covers “*the whole process from the procurement and storage of raw materials, through production, packaging, labelling and storage of the final product, to quality control and distribution, including internal audits*” (Sikora, 2015). Unlike in Switzerland, in the UK, GMP certification is not required for feasibility clinical trials (which measure only feasibility outcomes such as recruitment and retention of participants, but make no claims about efficacy), but it is required for full-scale clinical trials.

## 3 Why is it difficult to meet these requirements in the context of HFSs?

Conducting clinical trials with HFSs presents several challenges (summarised in Table 1).

**TABLE 1:** Summary of the current challenges for performing clinical trials with herbal food supplements.

Current situation	Proposed new standards
Quality of herbal food supplements (HFS) is unknown	Manufacturers should follow the ConPhyMP guidelines to provide comprehensive data on both the finished product and the starting plant material
Manufacturers are not held accountable for fraud because the quality of HFS is rarely checked	Report academic research findings highlighting low product quality to the relevant government agencies
	Implement regular random sampling by an independent government agency to verify that HFSs comply with legal requirements
Good manufacturing practice (GMP) certification is required for products used in clinical trials	Products that demonstrate adequate quality (and are already on the market) should be eligible for evaluation in clinical trials even without GMP certification

One major challenge concerns the quality and traceability of the plant raw materials. Regulatory authorities require that agricultural practices—including cultivation, harvesting, and primary processing—be thoroughly documented (Fürst and Zündorf, 2015). Herb suppliers must comply with Good Agricultural and Collection Practices (GACP) to ensure the authenticity and consistent quality of the starting material and reduce the risk of contamination by pesticides and heavy metals (van Breemen, 2015). However, the HFS supply chain often involves multiple intermediaries, making it difficult to identify the original supplier and verify GACP compliance and details of the chemical profile (Heinrich and Booker, 2016). In some cases, financial limitations may prevent some suppliers from upgrading their facilities to meet GACP standards (Schmidt, 2016).

Another challenge relates to the manufacturing process of HFS. Some companies are reluctant to disclose production details (Wright et al., 2022), partly because HFSs are difficult to patent (Kartal, 2007; Naumann, 2017). As a result, the extraction process is rarely described in natural products research (Heinrich et al., 2022). Standardizing herbal products is also problematic, since the composition of the raw plant material varies significantly depending on factors such as harvest season and time, temperature and soil conditions (Srinivasan, 2006). Furthermore, because therapeutic effects may not be attributed to a single metabolite or one class of metabolites, it is often difficult to define a suitable marker for quality control and standardization (Pferschy-Wenzig and Bauer, 2015). In many cases the actives remain unknown or poorly understood. While these issues can also affect HMPs, HFSs are subject to weaker quality control standards. This frequently leads to discrepancies between labelled and actual doses (Bourqui et al., 2025; Orhan et al., 2024). Performing RCTs with products of poor quality would have little value, as this would undermine the interpretation of the results (Wolsko et al., 2005).

Furthermore, HFSs can be marketed under food legislation without any GMP certification. Companies have no reason to obtain GMP certification, which demands substantial time and financial investment (Biagi et al., 2016; Yeung et al., 2008). However, this lack of GMP certification makes it impossible to conduct definitive clinical trials on these products.

## 4 Challenges in practice: cases from Switzerland and the UK

To illustrate the complexities surrounding clinical trials with a HFS and a GMP preparation developed for a RCT, we discuss two real cases, one in Switzerland and the other in the UK.

In Switzerland, we intended to conduct a randomised controlled clinical trial (RCT) with *Andrographis paniculata* (Burm.f.) Wall. ex Nees [Acanthaceae; aerial parts], traditionally used in Asia for acute respiratory tract infections. In Switzerland and Europe, it is only available as a HFS. This plant has one of the strongest evidence bases among herbs for respiratory infections (Hu et al., 2017). Despite its authorization and commercial availability in Switzerland as a HFS, sourcing a product suitable for clinical trials posed significant challenges. We contacted ten companies across Switzerland and Europe that manufacture HFS products derived from this plant, of which fewer than half were GMP-certified. Among those certified, one company initially expressed willingness to provide the product for the trial but was subsequently unable to complete the required pharmaceutical quality dossier. They could not disclose the provenance of the starting material and therefore could not attest that they were compliant with GACP. In addition, their quality control procedures did not meet the requirements set by the European Pharmacopoeia; their level of control was too low to ensure product safety and consistency. Independent testing commissioned from a third-party laboratory–the University of Geneva - found that the product was of low quality and, therefore, unsuitable for the clinical trial (Bourqui, personal communication). This led to quality control of a panel of internationally marketed *A. paniculata*-containing products, showing that the vast majority of products analysed were of low quality (Bourqui et al., 2025). So, although this plant is readily available to any consumer, we were ultimately unable to procure a product that met the regulatory standards required for clinical trial approval, hence putting an end to our efforts. The same issue was encountered in the UK. Although a feasibility trial has been completed, progression to a full trial is impossible until a GMP preparation is identified (Logue, 2020).

In the UK, we conducted a full-scale trial of *Arctostaphylos uva-ursi* (L.) Spreng. [Ericaceae; aerial parts] for acute uncomplicated urinary tract infections in women (Moore et al., 2019). Uva-ursi has a documented traditional use for urine infections dating back to the 13th century and is licensed in Germany. Although it was available over the counter in the UK, no GMP product was available on the market. Therefore, we decided to manufacture a GMP preparation for the trial. We obtained samples of the raw herb, reported to contain 20% arbutin, sourced from Russia and supplied by Martin Bauer GmbH & Co. KG (Vestenbergsgreuth, Germany), who ensure full traceability from plant to packaging. Samples of the raw herb were analysed via HPLC to determine the quantity of arbutin and free hydroquinone present, and results were compared to a Kew voucher specimen. It contained 20.046% arbutin and was re-tested 1 year later for stability and contained 20.998% (Trill, 2017). This plant material was then used to manufacture an extract in Germany by Finzelberg GmbH & Co., a GMP manufacturer, which was supplied by Temmler Pharma GmbH & Co. This was imported into the UK by Essential Nutrition Ltd. (a MHRA licensed GMP manufacturer) as we needed a qualified person (QP) to certify that each batch had been manufactured, imported, and released in compliance with GACP and GMP standards before it was supplied to the clinical trial sites. Essential Nutrition then encapsulated the herbal extract and produced placebo capsules. With a lack of previous dose-response studies, the trial dose had to be thoroughly investigated for the MHRA; this was based on monographs, herbal texts and small clinical studies. This whole process was excessively time-consuming and expensive, costing £92,500 to produce enough capsules to treat 382 patients (a cost of £242 per patient!) and taking over 2 years. Given these experiences, the team decided it would not follow this approach again for future trials of herbal medicines.

## 5 Discussion

Given that so few HFSs contain what is stated on their label, there needs to be more frequent quality controls of HFS, with penalties for companies who are selling sub-standard products. In the UK, the Trade Descriptions Act (1968) makes it illegal to provide false descriptions of products (The National Archives, 2025). In addition, according to the Food Supplements Regulations (2003) in England, the label must state the amount of any substance with a nutritional or physiological effect, based on the manufacturer’s measured average in the product. Comparable legal requirements exist in Switzerland. However, as the quality of HFS is hardly ever checked, the manufacturers are not held accountable for this. As there are so many food supplements on the market, it would be very challenging to test all of them on a regular basis. Random sampling is, however, both feasible and necessary. We argue that an independent government agency should take responsibility for such analyses. In Switzerland, the Federal Food Safety and Veterinary Office assigns this task to the cantonal enforcement authorities, who are expected to verify compliance of HFS with legal requirements through random checks (Office fédéral de la sécurité alimentaire et des affaires vétérinaires, 2024). Quality control performed as part of academic research could also contribute, if results are reported to relevant government agencies (e.g., Trading Standards in the UK). A consensus based statement, the ConPhyMP standards (Heinrich et al., 2022), provide a clear framework for manufacturers to supply the information needed to prove product quality and to ensure research reproducibility. Key data must include harvesting and collection methods, the plant part(s) used, the extraction process, and the standardization or quantification of marker compounds. These standards clearly define the minimum requirements for reporting.

Beyond issues related to product quality, additional challenges arise from the regulatory framework on the use of plants in HFSs. As the same plant may be sold as HFS and as HMP, it is challenging for lay consumers to distinguish between them, which can lead to misunderstandings about their use. The EU Claims regulation stipulates that health claims made on HFSs should only be authorized after a scientific assessment of the highest possible standard by the European Food Safety Authority (EFSA) (European Commission, 2020). However, in its evaluation of Regulation 1924/2006 on nutrition and health claims, the European Commission reported that no health claim relating to plants used in HFSs had received a positive EFSA assessment, mainly due to the lack of RCTs (European Commission, 2020). As a result, an “on-hold” list of health claims for plant was established. This regulatory ambiguity is problematic, as the continued use of “on-hold” claims on some HFS may mislead consumers into assuming that such claims have been scientifically validated and that the associated risks have been managed (European Parliament, 2023). Therefore, performing RCTs with HFS to assess these health claims is essential.

If HFSs meet the standards of quality and for reporting their composition, researchers should be permitted to evaluate them in clinical trials, without incurring the additional costs and bureaucracy of GMP. As the burden of custom-making a GMP formulation is excessively expensive and time-consuming, this essentially blocks any clinical trials on HFSs, although they are very widely used. Regulators need to consider (a) how they can enforce Trading Standards legislation on HFSs and (b) whether it is in the public interest to prevent clinical trials of HFSs, if these are of proven quality.

While herbs can be easily sold and consumed as HFSs, they remain difficult to evaluate through clinical research aimed at establishing their safety and efficacy. We argue for stricter enforcement of regulations to ensure that HFS contents match their labelling. At the same time, we recommend adapting the legal framework to better support clinical trials involving HFSs, for instance, by waiving the requirement for GMP certification.
